# Using time to investigate space: a review of tactile temporal order judgments as a window onto spatial processing in touch

**DOI:** 10.3389/fpsyg.2014.00076

**Published:** 2014-02-17

**Authors:** Tobias Heed, Elena Azañón

**Affiliations:** ^1^Department of Psychology and Human Movement Science, University of HamburgHamburg, Germany; ^2^Action and Body Group, Institute of Cognitive Neuroscience, University College LondonLondon, UK

**Keywords:** spatial processing, body schema, reference frame, remapping, multisensory integration

## Abstract

To respond to a touch, it is often necessary to localize it in space, and not just on the skin. The computation of this external spatial location involves the integration of somatosensation with visual and proprioceptive information about current body posture. In the past years, the study of touch localization has received substantial attention and has become a central topic in the research field of multisensory integration. In this review, we will explore important findings from this research, zooming in on one specific experimental paradigm, the temporal order judgment (TOJ) task, which has proven particularly fruitful for the investigation of tactile spatial processing. In a typical TOJ task participants perform non-speeded judgments about the order of two tactile stimuli presented in rapid succession to different skin sites. This task could be solved without relying on external spatial coordinates. However, postural manipulations affect TOJ performance, indicating that external coordinates are in fact computed automatically. We show that this makes the TOJ task a reliable indicator of spatial remapping, and provide an overview over the versatile analysis options for TOJ. We introduce current theories of TOJ and touch localization, and then relate TOJ to behavioral and electrophysiological evidence from other paradigms, probing the benefit of TOJ for the study of spatial processing as well as related topics such as multisensory plasticity, body processing, and pain.

## INTRODUCTION

The sense of touch is essential for many aspects of human function and cognition. Touch is intricately interweaved with the planning of our actions, with the perception of pain, with the defense of our body against physical threats, and, ultimately, with our sense of self. Among the different functions related to touch perception, its spatial processing – that is, where we perceive a touch to have taken place – has received considerable attention in psychology and neuroscience. In this review, we will explore some of these efforts, focusing on one specific experimental paradigm, tactile temporal order judgments (TOJ). This paradigm has proven particularly valuable for the investigation of tactile localization and its relationship to the many touch-related research topics, in particular when combined with changes in limb position. The most influential postural manipulation has been limb crossing. In fact, many experimental paradigms besides TOJ have relied on this manipulation, and, accordingly, the merits of limb crossing as an experimental manipulation for the investigation of touch will be extensively discussed.

In a typical TOJ task, participants are presented with two tactile stimuli, one to each hand, in short temporal succession. Participants’ task is to report which of the two stimuli came first. With uncrossed hands, human observers can resolve stimulus order accurately even at very short intervals (~30–70 ms), but performance becomes markedly impaired when the hands are crossed, with a larger time interval required between stimuli for correct performance (~120–300 ms, Yamamoto and Kitazawa, 2001a; Shore et al., 2002). In fact, the sequence of touches is often perceived in reversed order, indicating that the tactile events are systematically referred to the wrong hands (e.g., Yamamoto and Kitazawa, 2001a). This crossing effect in touch is thought to be due to a conflict between two spatial reference frames that are concurrently active. One reference frame is skin-based and, accordingly, somatotopically organized, and the other is external-spatial, possibly based on representations of visual space. Notably, the crossing effect is large in size, and it is reliable and stable, persisting even when the two tactile stimuli differ in frequency or duration (Roberts and Humphreys, 2008), and regardless of gender (Cadieux et al., 2010) and handedness (Wada et al., 2004), though the latter two can affect the size of the effect. Furthermore, the crossing effect persists when no time restrictions are imposed, and when only one stimulus order (e.g., right-hand first) requires a response, such as in a go/no go task (Roberts and Humphreys, 2008). The persistence of the TOJ crossing effect makes this paradigm particularly attractive for the investigation of touch localization.

We will first inspect the TOJ task and the processes it is thought to involve. We will establish different ways with which TOJ performance can be measured, and scrutinize the paradigm’s merit in investigating spatial processing. We will then give an overview over current theories that attempt to explain crossing effects, both generally, and specifically for TOJ, and discuss the time course of the localization process. Once these aspects have been covered, we will then show how the paradigm has been helpful in the investigation of several areas of research, including the time course and reference frames involved in tactile localization, multisensory plasticity and integration, bodily awareness and its disorders, and pain perception.

## MEASURING THE TOJ CROSSING EFFECT

**FIGURE 1:**
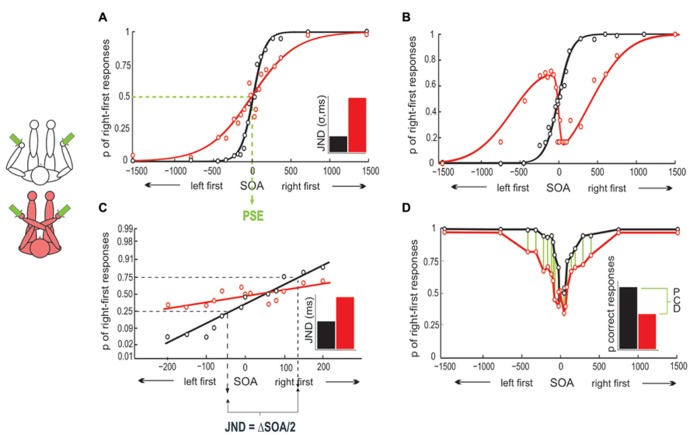
**Different analysis options for TOJ. (A)** Typical single participant result of uncrossed and crossed hands TOJ. With uncrossed hands, the psychophysical curve is steeper than with crossed hands, indicating the performance advantage for the uncrossed posture. The PSE is the SOA at which left and right stimuli are equally often perceived to have occurred first. The just noticeable difference (JND) corresponds to the standard deviation of the Gaussian fit in this panel. It denotes the SOA at which participants judge stimulus order correctly in 84% of trials. The inset illustrates the JND difference for uncrossed and crossed postures. **(B)** Hypothetical example of N-shaped response probability profile in a crossed posture. **(C)** Probit analysis of the data in **(A)**. Right-first response proportions are linearized by probit transformation and regressed thereafter. The crossing effect is evident in the difference of slopes between conditions. The JND is derived by projecting the 25 and 75% right first probabilities onto the SOA axis. **(D)** Data in **(A)** can be plotted as proportion-correct rather than right-first. The crossing effect can then be assessed as the proportion correct difference (PCD, proportion correct difference, see Cadieux et al., 2010) in the two postures.

Temporal order judgment performance has been assessed with an unusually high number of different measures (see **Figure 1**), including measures of sensitivity and bias, as well as reaction time (RT). Crossing effects (i.e., differences in performance between crossed and uncrossed postures) have been observed with all of these measures.

### MEASURES OF SENSITIVITY

TOJ are typically assessed at several stimulus onset asynchronies (SOA), often in the range of 15–200 ms (e.g., Shore et al., 2002), but sometimes of up to 3000 ms (e.g., Yamamoto and Kitazawa, 2001a; Heed et al., 2012). By convention, left-first SOA are denoted as negative, and right-first SOA as positive; thus, an SOA of -50 ms indicates that the left stimulus led the right by 50 ms.

At each SOA, the percentage of right-first responses is used as a measure of performance. When plotted, performance resembles a typical psychophysical, S-shaped curve, which can be fitted reasonably well with cumulative Gaussian and logistic functions (See **Figure 1A**). The standard deviation of the Gaussian fit has been used as a summary statistic, and denotes the SOA at which participants judge stimulus order correctly in 84% of trials (e.g., Yamamoto and Kitazawa, 2001a; Azañón and Soto-Faraco, 2007). This time interval is referred to as the just noticeable difference (JND, **Figure 1A**). Graphically, an increase in the Gaussian’s standard deviation results in a shallower rise of the *S*-curve. Thus, the smaller the JND (as expressed in the standard deviation), the steeper is the curve, and the better is performance.

A different approach to analyze TOJ is to linearize the S-shaped performance curve by probit-transforming right-first response probabilities at each SOA (e.g., Shore et al., 2002; Schicke and Röder, 2006; see **Figure 1C**). This approach has the advantage that linearization of response values allows the use of regular regression analysis. However, the disadvantage is that only short SOA can be analyzed with probit transformation. This is because the psychometric functions asymptote at higher SOA, and as a consequence, probit transformation is not adequate to analyze large SOA (in psychometric fitting, two additional model parameters fit the upper and lower asymptotes, see Wichmann and Hill, 2001; Yamamoto and Kitazawa, 2001a; Roberts and Humphreys, 2008). The slope of the regression line can be interpreted in analogy to the Gaussian’s rise, with a steeper slope indicating better performance.

When responses are not analyzed with a Gaussian fit, the JND cannot be derived from a model parameter. Instead, the data points of the slope at which the proportion of right-first responses is 25 and 75%, respectively, are projected onto the SOA axis (Shore et al., 2002; see **Figure 1C**). The SOA between these two projections, divided by 2, is then referred to as JND and denotes the SOA at which the two tactile stimuli must be presented for the participant to make 75% correct responses^1^. Recall that the JND of the Gaussian fit indicated a correctness level of 84%; accordingly, the JND computed from the two analysis approaches are not directly comparable.

Crossing effects have also been assessed by comparing the cumulated percentage of correct responses over all SOA in uncrossed and crossed conditions (Cadieux et al., 2010, see also Heed et al., 2012, see **Figure 1D**); this measure has the advantage of being free of the assumption that the response profile across SOA follows a specific distribution (as is assumed by both psychometric function fitting and probit transformation), but, as opposed to the previous methods, it is blind to differences between SOA. Furthermore, percentage correct scores are the measure of choice when only one or two SOA are used (Roberts and Humphreys, 2008; Hermosillo et al., 2011), as curves and lines cannot be estimated in this case. 

### N-SHAPED RESPONSE CURVE

An unusual finding pertaining to TOJ is that some participants show systematically reversed (“flipped”) responses for short SOA in crossed postures. As a consequence, their response curves are N-shaped rather than S-shaped (Yamamoto and Kitazawa, 2001a; Azañón and Soto-Faraco, 2007; see **Figure 1B**). It is unknown whether participants displaying N-shaped response curves process TOJ differently than S-type participants, or whether their response pattern is an extreme variant of systematic errors observed in the reduced steepness of S-curves in crossed conditions in other participants. Some studies have, therefore, excluded N-shape participants (Kóbor et al., 2006).

When analyzed with probit slopes (which include only short SOA, that is, the descending leg of the N), N-shapes result in negative slopes and can be included in a group analysis. Alternatively, data can be fitted with the “flip” model (Yamamoto and Kitazawa, 2001a). This model uses different functions to fit performance in uncrossed and crossed postures. For uncrossed postures, which are reliably S-shaped, data are fitted with a cumulative Gaussian. For crossed postures, two normal curves (i.e., “non-cumulative” Gaussians) are added to the cumulative Gaussian fitted to the *un*crossed condition. The two additional Gaussians account for the flip and are proposed to reflect a specific, additional process prompted by limb crossing. Importantly, the model fits both S- and N-shaped response curves for crossed conditions and does not, therefore, need to posit that there are processing differences between N and S-type participants. However, the model requires five free parameters and, thus, requires a large amount of SOA.

### MEASURES OF BIAS

S-shaped response curves are not only defined by the standard deviation – a measure of sensitivity –, but additionally by their mean, that is, the SOA at which a participant perceives the two stimuli to be simultaneous and, accordingly, responds “right first” and “left first” equally often. In psychophysics, this SOA is referred to as the point of subjective simultaneity (PSS) or the point of subjective equality (PSE), and denotes a bias toward one or the other response. In TOJ, one would expect the PSS for two stimuli to be 0, reflecting that participants perceive simultaneity when the stimuli are indeed presented simultaneously (see **Figure 1A**). However, the PSS may differ from zero for a number of reasons, for example due to differences in neural transmission speeds when stimuli are presented to different body parts, or due to handedness (Wada et al., 2004). Changes of the PSS have been relevant especially in clinical context (e.g., Moseley et al., 2009). Note, that the bias is independent of sensitivity. Thus, a change of the PSS is independent of a change of the slope. This expresses that participants may be biased toward one of the two stimuli, but be uncertain about their response only within a small range of SOA.

### REACTION TIME

Crossing the hands affects not only response accuracy, but also RT. As for the proportion of right-first responses, RT can be assessed separately for each SOA, or be cumulated across all SOA. It is generally found that RT decreases with increasing SOA, resulting in a roof-like RT curve. When analyzed by SOA, RT differences between postures (i.e., uncrossed vs. crossed) are sometimes greater at longer than at shorter SOA (Yamamoto and Kitazawa, 2001a; Heed et al., 2012), but in any case, faster responses are found for uncrossed hands. Note, that participants are usually asked to respond as accurately as possible, without emphasis on speed. In principle, RT effects may be different if speed were stressed.

The obvious disadvantage of the plethora of measures used for the TOJ paradigm is that comparison across studies can be difficult. A systematic comparison of the advantages and disadvantages of each measure, for example in terms of sensitivity, fitting error, etc., has not been published. However, one recent study compared a large part of the above-mentioned measures for three experiments and found largely consistent results across measures for comparisons of uncrossed and crossed conditions (Heed et al., 2012). However, crossing effects were more reliable for accuracy than for RT, with some crossing effects not evident in the latter.

## CROSSING EFFECTS: SPECIFICITY FOR SPATIAL PROCESSING

### STIMULATION OF NON-HOMOLOGOUS BODY PARTS

**FIGURE 2:**
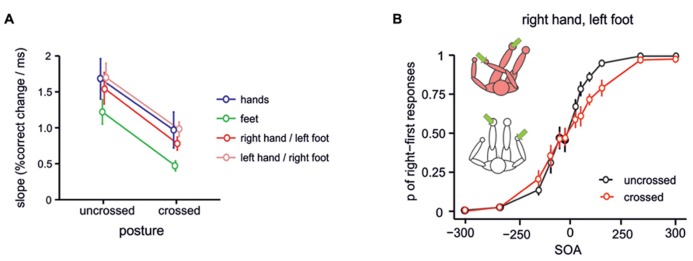
**TOJ crossing effects for stimuli presented to hands and feet.** Crossing effects are evident for TOJ between different limbs, suggesting that TOJ effects are not due to activation of homologous regions in primary somatosensory cortex, but stem from stimulus coding in a higher-level spatial representation. **(A)** Probit slopes for uncrossed and crossed conditions with different combinations of hand and foot stimuli. A crossing effect was present for all combination of stimulated limbs. **(B)** TOJ performance for stimulation of the right hand and left foot. Figure modified from Schicke and Röder (2006), Copyright (2006) National Academy of Sciences, USA.

When stimuli are applied to the two hands in the TOJ paradigm, one might suggest that the crossing effect arises because the homologous regions of primary somatosensory cortex (SI) are activated in short succession. Because there is crosstalk between homologous regions of SI, mediated by connections crossing the corpus callosum (Iwamura et al., 2001; Jung et al., 2012), bilateral stimulation may hinder a sensitive comparison of the two tactile stimuli. However, TOJ crossing effects are also evident when the two tactile stimuli differ in characteristics like frequency or duration (Roberts and Humphreys, 2008). Individualizing stimulus characteristics might be expected to lead to differences in activity in primary somatosensory cortices. Yet, information is still transmitted across the corpus callosum even when stimuli are clearly different. Hence, this transferred information may still be the cause of stimulus confusion in TOJ. To address this possibility, TOJ stimuli have been presented to non-homologous limbs, assuming that the body part-specific cross-callosal connections should then not play a role in stimulus comparisons. Crossing effects were comparable when homologous fingers (e.g., the two index fingers) and non-homologous fingers of the two hands (e.g., index vs. little finger) were stimulated (Shore et al., 2002; Heed et al., 2012). Maybe more compellingly, when a tactile stimulus pair was presented to one hand and the opposite side’s foot, crossing the arm over the leg impaired performance in a similar manner as when two hands or two feet were stimulated in crossed postures (Schicke and Röder, 2006, see **Figure 2**). Thus, a crossing effect was evident although the two tactile stimuli were applied to entirely different body parts. Because callosal connections between the SI of the two hemispheres are specific to homologous regions (Jung et al., 2012), these results suggest that TOJ crossing effects are not a result of lateral connections in SI.

### VARIATION OF RESPONSE MODALITY

In tactile TOJ paradigms, responses are often given with the limbs that receive tactile stimulation, that is, with a crossed limb in crossed conditions. The main reason for this practice is that this stimulus-response mapping can be instructed without the use of the terms “left” and “right.” Any other mapping (e.g., when using foot responses) requires specification of what is meant by left and right – the hand (anatomical coding) or space (external coding). However, an obvious criticism of this response mode is that crossed postures may provoke higher RT and higher error rates because they are unusual and uncomfortable. Yet, hand posture does not seem to influence RT in simple detection tasks in which participants simply respond as fast as possible when a stimulus is perceived, and do not have to make a choice about the stimulus. For instance, hand crossing did not affect performance is such a task with the use of visual stimuli (Anzola et al., 1977; Nicoletti et al., 1984). Analogous results have been obtained in touch: detection (as opposed to localization) of tactile stimuli were unaffected by hand posture (Badde et al., 2012). In contrast, when a choice had to be made as to whether the touch was presented to the right or the left hand, a crossing effect was observed. Similarly, when TOJ were made about two visual stimuli occurring near the hands, there was either no crossing effect at all (Yamamoto and Kitazawa, 2001a), or it was substantially reduced to a difference in JND of just 5 ms between postures (compared to 90 ms for tactile stimulation, Shore et al., 2002). Moreover, the deficit not only occurs when responses are given with the stimulated finger, but also when participants respond with a foot pedal (Yamamoto et al., 2005; Heed et al., 2012), respond verbally (Pagel et al., 2009; Hermosillo et al., 2011), or by looking at either a neutral target or toward the limb that was stimulated first (Yamamoto and Kitazawa, 2001a; Pagel et al., 2009). In contrast, when the order of two tactile stimuli must be determined with respect to a non-spatial criterion, for example, stimulus duration or vibration frequency, then no crossing effects are observed (Roberts and Humphreys, 2008), presumably because tactile localization is no longer required. All these findings suggest that the TOJ crossing effect is intimately related to touch localization in external space, and is not due to difficulties in responding with crossed hands.

### SPATIAL EFFECTS WITH UNCROSSED LIMBS

It is of note that effects of hand posture are also observable independent of hand crossing. For example, TOJ are slightly better when the hands are placed far apart rather than close together (Shore et al., 2005) whether or not the hands are crossed (Roberts et al., 2003). This effect is present even when the separation between the limbs is illusory, for example when the visual appearance of arm posture is manipulated by means of a mirror reflection while arm posture is actually kept constant (Gallace and Spence, 2005). Although such posture effects achieved without limb crossing result in much smaller effects than crossing manipulations, their existence nonetheless suggests that integration of skin location with body posture is indeed a general principle of tactile localization, and is not a special case pertaining to crossed limbs alone (see Azañón et al., 2010b).

Finally, one might argue that crossing effects are due to perceptual processes unrelated to localization, such as posture itself, that is, crossing any body part would influence perceptual judgments of any other body part. Several experimental findings argue against this view. When TOJ are made about stimuli at the tip of sticks, a crossing effect is evident also when the sticks are crossed while the hands remain uncrossed (Yamamoto and Kitazawa, 2001b). In this situation, body posture is unchanged, indicating that the crossing effect cannot be due simply to postural factors. Furthermore, in a recent study, stimuli for the TOJ task were delivered to the little fingers while the index fingers were crossed (Badde et al., 2013). TOJ for the little fingers were entirely unaffected by the index fingers’ posture, again suggesting that introducing a crossing manipulation affects the spatial processing of touches to the crossed body parts, but does not affect more general aspects of touch processing, e.g., due to discomfort or general confusion.

## THEORIES OF TOUCH REMAPPING

Four distinct theoretical approaches have been proposed to account for TOJ crossing effects.

### SPACE–TO–BODY PROJECTION ACCOUNT

The first account, put forward by Kitazawa and colleagues (Yamamoto and Kitazawa, 2001a; Kitazawa, 2002), assumes that a comparison of tactile stimuli requires conscious access to their representation. Most importantly, conscious perception is suggested to rely on an external spatial reference frame. Specifically, a stimulus is first perceived in space and then projected back onto the skin location whence it was perceived. Thus, in this account remapping is directed from the external to an anatomical location. For example, a stimulus to the left crossed hand is perceived as a right spatial event and is then assigned to the left hand, which currently occupies that spatial location. Remapping is assumed to take about 300 ms when the hands are crossed, based on the systematic reversals observed when two tactile stimuli are presented at short SOAs (Yamamoto and Kitazawa, 2001a). As long as remapping has not been performed, the brain is suggested to rely on a default posture of the body, according to which each hand is located in its regular hemispace. In the TOJ task with crossed hands the second stimulus, in the case of short SOA, is then thought to arrive before remapping has been completed, leading to erroneous assignment of the tactile stimulus to the wrong hand based on the default posture, rather than based on the remapped posture (see Azañón et al., 2010b and Longo et al., 2010, for further considerations about the default posture).

### APPARENT MOTION ACCOUNT

A second account, put forward by Kitazawa and colleagues some years after their first account (Kitazawa et al., 2008; Takahashi et al., 2013), assumes that ordering stimuli in time is achieved by integrating single stimuli into a motion signal. Similarly to visual apparent motion, tactile stimuli in the TOJ task are suggested to give rise to an illusory motion percept. According to this account, the TOJ is based on the direction of motion. The stimulus that occurred earlier according to the motion percept is judged as having occurred first. In the case of crossed TOJ, each stimulus location is initially projected to the wrong hand (analogous to the authors’ first account). At short SOA, the motion signal is therefore constructed with an inverted direction vector, leading to erroneous TOJ responses. Motion stimuli have been found to affect TOJ (Craig, 2003; Craig and Busey, 2003; Sanabria et al., 2005; Shibuya et al., 2007; Kitazawa et al., 2008), suggesting that motion signals may indeed be important for TOJ. Furthermore, tactile apparent motion was found to be strongest for those SOA at which responses are most likely flipped for N-shape participants (Takahashi et al., 2013).

### SPATIAL CONFLICT ACCOUNT

The third account, put forward by Shore and colleagues (Shore et al., 2002; Cadieux et al., 2010), assumes that a tactile stimulus is initially represented according to its somatotopic location on the skin and then remapped into external coordinates. Afterward, the two spatial representations, each based on a different reference frame, are concurrently available. In the TOJ task, this concurrent availability leads to conflict, because each stimulus is now represented with both left and right characteristics. Crossing effects in terms of higher RT are then proposed to be due to the time required to resolve this conflict. Crossing effects in terms of higher error rates are attributed to confusion due to conflicting information, and the cognitive effort to resolve the conflict.

### SPATIAL INTEGRATION ACCOUNT

The fourth account, put forward by Badde et al. (in press), is similar to that of Shore and colleagues in that it assumes that somatotopic and external spatial reference frames are concurrently active. However, it is assumed that a location estimate is computed for each stimulus by integrating all sources of information (here, somatotopic and external) with specific top-down modulated weights. A role for top-down modulation has been inferred from the finding that memory load modulates crossed hands performance, suggesting that tactile remapping might not proceed entirely automatically (Badde et al., in press). According to Badde et al. (2012) integration is carried out in any tactile task, and should, therefore, result in crossing effects even in tasks that involve only a single tactile stimulus. Importantly, weights are not adjusted according to posture, but only according to task demands. That is, this account explicitly proposes that limb crossing leaves the manner in which the different reference frames are integrated unchanged. Errors in this account are instead attributed to the probabilistic outcome of the spatial integration process. The large size of the crossing effect in the TOJ task is attributed to a reduction of certainty about the location of the first stimulus due to the integrated location estimate of the second stimulus. A possible implementation of the integration across reference frames has been proposed in a different context (Buchholz et al., 2012). These authors suggested that excitatory and inhibitory interactions within spatial maps, known to be at work, for example, in the superior colliculus, may account for changes of behavioral performance in the orientation of coordinated eye-head movements to visual-tactile stimuli across different hand postures.

### COMMON ASPECTS OF ALL THEORETICAL ACCOUNTS

Several aspects are common to all four theoretical accounts. First, all accounts posit transformation processes for tactile stimuli between somatotopic and external spatial coordinates. This aspect is probably the most important feature of the TOJ paradigm. Given that non-spatial explanations of the TOJ crossing effect (discomfort, inexperience, etc.) have been discounted, the presence of a crossing effect is therefore interpreted as an indicator that spatial remapping does indeed take place in a given experimental situation. This conclusion is independent of the theoretical approach the experimenter may favor. Second, all four accounts posit the involvement of (at least) two spatial representations in touch localization. Kitazawa and colleagues propose that a representation of a default posture is available for use when the calculation of the skin coordinate of the stimulus has not been determined. The other accounts propose that stimuli are initially represented with respect to the skin, and are recoded into an external spatial location. In contrast, the accounts differ in how they explain the performance deficit observed in TOJ: according to Kitazawa and colleagues, errors in the crossed posture are due to the use of the default representation, which introduces a conflict with the limbs’ true posture. Shore and colleagues as well as Badde and colleagues posit that TOJ performance deficits during crossing result from resolution of conflict (Shore) and from integration of information (Badde).

In sum, although different theoretical proposals have been put forward to account for TOJ crossing effects, they all agree in that they interpret the existence of crossing effects as an indicator of spatial remapping, as well as an indicator of the use of external spatial coordinates in touch.

## THE TIMING OF TACTILE REMAPPING

**FIGURE 3:**
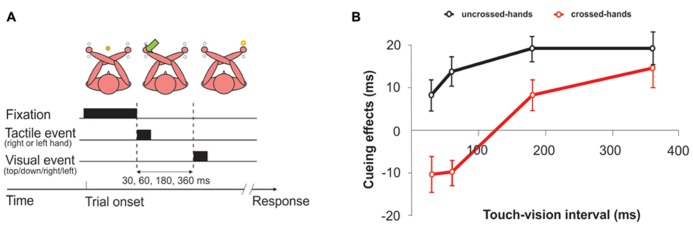
**Cueing effects between touch and vision.** An inversion of spatial cueing effects of touch on vision was observed when the hands were crossed. These results suggest that touch is initially remapped on the basis of its anatomical representation before it is referred to an external location. **(A)** Typical trial with crossed hands. Participants were asked to judge the position of the light (up or down), regardless of the side of presentation and the location of the preceding tactile cue. **(B)** When the interval between tactile cues and visual targets was less than 60 ms, spatial cueing effects appeared to be determined by somatotropic representations, as responses to the visual targets were faster in opposite cue-target side trials (anatomically congruent but spatially incongruent) than in same-side trials. The pattern reversed after about 200 ms, so that tactile cues produced a facilitation of targets presented at the same external location. No differences were found with uncrossed hands. Figure modified from Azañón and Soto-Faraco (2008a), with permission from Elsevier.

The suggestion that remapping into external spatial coordinates is a time-consuming process raises the question of which time course this process may take. In their initial study, Yamamoto and Kitazawa (2001a) found performance with crossed hands to be similar to performance with uncrossed hands when stimuli were approximately 300 ms apart. Accordingly, they suggested this duration as an estimate for the duration of the remapping process. Several studies have since been dedicated to this issue. In one study, participants had to judge the elevation of a visual stimulus (up vs. down), which could be presented in the left or the right hemifield (Azañón and Soto-Faraco, 2008a, b; see **Figure 3**). A spatially non-predictive tactile cue, delivered to one of the hands, preceded the visual stimulus at different intervals. When the hands were crossed, responses to the visual stimulus were faster when it occurred on the anatomically same side as the tactile cue (that is, in the opposite side of space) when the SOA between the two stimuli was short (<60 ms). In contrast, responses were faster for the external-spatial side of the tactile cue when the SOA between tactile cue and visual stimulus was longer (360 ms). This result suggests that, initially, the brain has access only to the anatomical coordinate of the touch, but that the external coordinate becomes available some time between 60 and 360 ms. A change of reference frames has also been demonstrated for the execution of saccades to single tactile stimuli (Groh and Sparks, 1996; Overvliet et al., 2011). When the tactile stimulus was delivered to crossed hands, saccades regularly started in the direction of the wrong hand and turned around to the correct hand in mid-flight. This turn was evident, on average, 284 ms after initiation of the saccade. Because the time needed for the actual motor preparation was estimated, based on findings in monkeys, to be in the range of 95 ms (Overvliet et al., 2011), the authors concluded that remapping must be completed already around 190 ms after stimulation. The estimate given for completion of remapping was even lower in a study that employed stimulation of the hands and feet while measuring somatosensory evoked potentials (SEP; Heed and Röder, 2010). This study factorized anatomical and external distance between attended and stimulated locations by asking participants to attend to one limb while tactually stimulating another limb with a single stimulus. External distance was manipulated by changing hand posture, such that the hands were spatially close either to the respective foot of the same body side (with uncrossed hands) or to the respective opposite foot (with crossed hands). The SEP in the time range between 100 and 140 ms was modulated both by anatomical distance as well as by external distance between attended and stimulated location. This result implies that external coordinates can be available already at this time point, given that attention was partly directed according to external spatial criteria. This time estimate was confirmed in a setting which did not rely on attentional manipulations, but compared SEP to single tactile stimuli when the hands were uncrossed vs. crossed (Rigato et al., 2013). Finally, a significant difference between uncrossed and crossed postures was evident over the left temporal scalp at even earlier stages of tactile processing (70–90 ms post-stimulus) independent of the hand at which the stimulus had occurred (Soto-Faraco and Azañón, 2013). Moreover, the size of the ERP posture effect was positively correlated with participants’ TOJ crossing deficit measured in the same session. Thus, the larger the difference due to posture in somatosensory processing (indexed by the SEP), the larger was the crossed-hands deficit in the TOJ task.

There is currently no theoretical account that integrates these different findings regarding the timing of tactile remapping (though see Soto-Faraco and Azañón, 2013 for a consideration of different deflections of the SEP in the context of remapping). Yet, the picture emerging from these studies is consistent, in that tactile information appears to be used in the original, somatotopic reference frame, but becomes available in the external reference frame rather quickly, probably within the first 100 ms, but maximally 190 ms post-stimulus.

## TYPES OF REFERENCE FRAMES INVOLVED IN TACTILE LOCALIZATION

The automatic recoding of touch into external coordinates may provide important advantages for the processing of tactile information. On the one hand, it may allow efficient integration of spatial information derived from touch with information from the other senses. On the other hand, an external spatial coordinate may allow rapid orienting and movement toward the tactile event.

Vision has been suggested to dominate the other senses in spatial processing under normal circumstances (Alais and Burr, 2004), due to its high acuity when compared to that of audition and touch. Indeed, it plays a dominant role in sensorimotor coordination as the location of reaching targets is often encoded in eye-centered coordinates, irrespectively of the target modality and the effector to be used (Batista et al., 1999; Cohen and Andersen, 2000; Pouget et al., 2002). Accordingly, the external coordinates used by the tactile system may be visual in nature. Such recoding would be attractive from a multisensory perspective, effectively converting any touch we perceive into the coordinates used by the visual system for immediate integration.

### DEVELOPMENTAL ASPECTS: BLIND INDIVIDUALS

**FIGURE 4:**
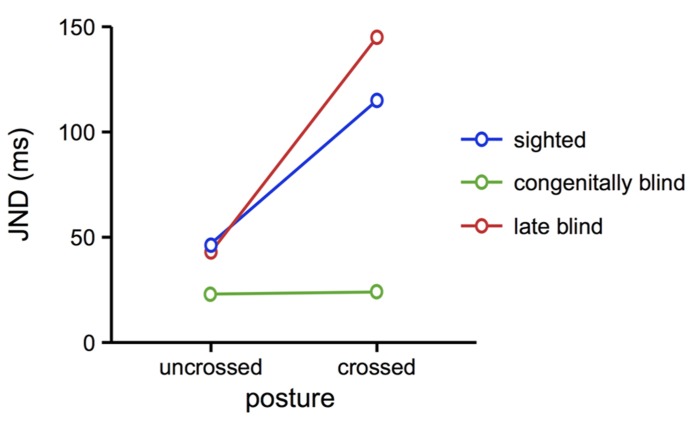
**TOJ performance of blind compared to sighted individuals.** Congenitally blind individuals performed equally well with uncrossed and crossed hands. In contrast, late blind individuals and sighted participants showed the typical crossing effect, with larger JND in the crossed posture. These results suggest that the automatic use of external coordinates in touch localization depends on visual development during ontogeny. Figure modified from Röder et al. (2004), with permission from Elsevier.

Even though remapping does occur in absence of vision, for instance when locating tactile stimuli in the dark or with a blindfold (Kóbor et al., 2006; Schicke and Röder, 2006), several studies suggest that tactile remapping is closely related to the development of the visual system during ontogeny. For instance, congenitally blind participants were unaffected by crossing the hands when performing a TOJ (Röder et al., 2004, see **Figure 4**). Strikingly, people who had turned blind later in life performed just like the sighted, and showed a marked crossing effect. Furthermore, a man born with bilateral cataracts and, thus, functionally blind, and whose vision was surgically restored at age 2, did not exhibit a crossing effect (Ley et al., 2013). Even more, this man did use external coordinates for the representation of touch in a task that involved bimodal, visual, and tactile, stimulation. These results suggest a pivotal role for the visual system during early life for the development of coordinate transformations in touch: if vision is available after birth, then the default use of an external reference frame is established and remains intact, even if vision is lost at a later point in time. In contrast, when vision is not available after birth, then the tactile system does not seem to integrate an external reference frame as a default source of spatial information, even if vision becomes available later. At least if vision is restored early on, then the use of external coordinates in touch can be established for specific situations, presumably predominately those involving the integration of touch with vision.

### DEVELOPMENTAL ASPECTS: CHILDREN

The finding that late blind individuals seem to use external coordinates in touch processing just like the sighted bears the question of when during ontogeny this processing feature develops. To this end, children between the ages of 5 and 10 were tested with the TOJ paradigm (Pagel et al., 2009). A crossing effect was not observed in the youngest children, up to about 5½ of age. After this age, a crossing effect was evident in some children, indicating that they used external coordinates to localize touch. At the age of about 8½, a crossing effect was seen in all tested children. The fact that some children did not show a crossing effect after the age of 5½ suggests that the integration of different reference frames may develop over an extended period of time, consistent with findings about the development of multisensory integration of touch with vision (Röder et al., 2013). Furthermore, it is important to stress that these TOJ results do not speak to the existence of an external reference frame in yet younger children. Rather, they suggest that its automatic use in touch does not start before age five. In contrast, that external coordinates are available for touch has been demonstrated for 10-month-old babies (Bremner et al., 2008). When they received a touch to crossed hands, their manual responses toward the stimulus were more often correct than not, indicating that stimulus location must have been coded in external spatial coordinates. However, these responses occurred only in some trials and several seconds after stimulus application, suggesting that the recoding of touch location into external coordinates is not yet automatic and efficient at this age.

### REPRESENTATION OF SPACE OUTSIDE THE VISUAL FIELD

These studies suggest that extensive visual experience during the first years of life might lead to crossmodal links between touch and vision that are used by the remapping system to encode touch in external space. This is probably related to the finding that the crossing effect was weaker when the hands were crossed behind the back, where no prior visual experience can lead to the configuration of visuotactile representations (Kóbor et al., 2006). A second study even found similar TOJ performance in front and back space, suggesting that the external coordinates used in touch cannot be solely related to vision, but must involve other reference anchors. It has been suggested that external coordinates in regions we cannot access directly through vision may be built up by the motor system (Heed and Röder, 2012). Imagine someone tipping you on the shoulder from behind. Your reaction will be to turn your upper body around to direct your eyes toward the person who touched you. A reference frame instrumental to such behavior would be one related to the movement necessary to reach a given location. Intriguingly, an ERP study of touch processing for stimuli in front of and behind the body supports this idea (Gillmeister and Forster, 2011). Stimuli were delivered to the two hands held close together or far apart. Participants had to detect infrequent target stimuli among the stimulus stream presented to the two hands. For each trial, a cue indicated where the stimulus would most likely occur. When the hands were positioned in front of the body, the effect of cueing was larger when the hands were held far apart than when they were held closer together, presumably because the greater spatial distance between the hands supported the selective attentional deployment to one hand (Eimer et al., 2004, see also Driver and Grossenbacher, 1996; Lakatos and Shepard, 1997; Soto-Faraco et al., 2004; Shore et al., 2005). Conversely, the ERP attention effect for stimuli behind the back was greater when the hands were held together than apart. The authors suggested that this result implies a spatial representation that “warps” around the body, much like one might expect from a representation based on movement.

The importance of movement planning for tactile localization is further highlighted by an experiment in which participants made TOJ in the context of hand movements. In each trial, participants adopted an uncrossed or crossed posture, and had to execute a movement with the two hands, to end in an uncrossed or crossed posture. Tactile stimuli were presented shortly before the movement was executed, that is, during the movement planning phase (Hermosillo et al., 2011). When the hands were uncrossed, but a crossing movement was planned, TOJ were impaired. In contrast, when the hands were crossed, but an uncrossing movement was planned, TOJ were ameliorated. These effects were stronger the closer the stimuli were presented to movement initiation and, therefore, the later they were presented in the motor planning process, suggesting a link between movement planning and the weight assigned to the future hand posture in judging tactile location. The authors suggested that the influence of motor planning on touch localization may be mediated by efference copy of the motor commands.

### INFLUENCE OF VISUAL MOTION ON TACTILE LOCALIZATION

Yet another demonstration of the importance of vision on touch remapping was given by adding visual location information in a tactile TOJ task. Two tactile stimuli were presented, one to each middle finger, in short succession. With each tactile stimulus, a visual stimulus was projected onto one of the middle fingers (Kitazawa et al., 2008). The order of the visual stimuli was either identical to the tactile stimuli, or reversed. When the spatial direction of visual stimuli was incongruent with that of tactile stimuli, many subjects reported inverted judgments, that is, they took visual information into account although it was task-irrelevant. A similar effect was evident when tactile stimuli were applied to three adjacent fingers on a single hand (Shibuya et al., 2007). In this latter study, simultaneous visual stimuli could occur in 9 locations, arranged as a square and projected on top of the hand. When the hand was directed away from the body, the effect of the visual stimuli was present when they were arranged from left to right. When the hand was rotated by 90°, the effect of the visual stimuli was present when they were arranged from top to bottom. Thus, the effects of vision on touch were mediated in an external reference frame.

In sum, many findings suggest a pivotal role of vision for touch localization. This effect is evident in two very different aspects. First, touch localization appears to develop differently when the visual system is present than when it is not, as in congenitally blind individuals. Second, when vision has developed normally, it not only provides a spatial reference frame for touch, but, in addition, strongly affects tactile localization by providing spatial information which appears to be integrated into the tactile location estimate. Nevertheless, the external reference frames on which the tactile system relies appear not to be exclusively visual, as demonstrated by the use of external reference frames in regions that are inaccessible to the visual system, and the effects of movement planning on tactile localization. These latter results suggest that external coordinates in touch may be determined, in part, by the motor system.

## FLEXIBILITY IN THE USE OF DIFFERENT REFERENCE FRAMES

The fact that late blind people show a crossing effect in TOJ many years after they have become blind seems to imply that the way the brain integrates information from the different reference frames is rather rigid. This implication is at odds with many other findings about the principles by which the brain integrates information across the senses. For example, haptic information is regarded more when the quality of visual information is degraded (Ernst and Banks, 2002), and such effects are observable between blocks of an experiment, that is, over short time scales. Such weighting of information from different sources is often near-optimal, in the sense that the importance given to a source of information is closely related to its reliability (Alais and Burr, 2004).

### BAYESIAN CALIBRATION OF LOCALIZATION

Following up on these principles, Miyazaki and colleagues (Miyazaki et al., 2006) manipulated the frequency with which the left and the right stimulus occurred first in a TOJ experiment with uncrossed hands. The distribution of SOA was biased toward one of the hands. This shift in SOA distribution led participants to adjust their responses such that the PSS – the SOA at which participants’ responses chose both hands with equal probability – was shifted toward the peak of the prior distribution. Formulated differently, when the distribution of SOA was biased toward one side, participants biased their response to report the stimulus of that side to have occurred first. This adjustment behavior is consistent with participants calibrating their responses in a Bayesian manner by adjusting their prior, rather than recalibrating the perceived time across hands by shifting the PSS as if the mean SOA of the shifted distribution were zero. Thus, such adjustment of responses probably does not reflect an alteration of perception. Rather, the change in response appears to reflect a strategic choice about how to integrate different sources of information for the choice of response (Smeets et al., 2006). Further underlining this interpretation, it has recently been demonstrated that several priors for the TOJ task can be acquired concurrently by setting different contexts, as for example a different color cue for each prior on the computer screen (Nagai et al., 2012).

### SHORT-TERM PLASTICITY: TOUCH LOCALIZATION AS WEIGHTED INTEGRATION

To test whether weighting is also applied during the integration of anatomical and external reference frames in touch localization, Badde et al. (2012) used a modified TOJ paradigm: in a first experiment, participants performed normal TOJ. In a second experiment, stimulation was identical to the TOJ paradigm, but participants were instructed to respond to the location of the first stimulus and ignore the second stimulus. Finally, in a third experiment participants were presented with only a single tactile stimulus. The task was identical as in their second experiment, that is, to respond to the location of the stimulus. Hand crossing affected all three tasks. Thus, both top-down information (i.e., the change of task instructions from experiment one to experiment two) as well as bottom-up information (i.e., a change in stimulation from experiment two to experiment three) affected the crossing effect. Probabilistic modeling suggested that these changes were accounted for by the weighing of anatomical and external spatial information during their integration. The model did not need to recur to the assumption that crossing deteriorated the quality of sensory information. These modeling results therefore imply that crossing effects, including those found in TOJ experiments, result from the brain’s strategy to derive a location estimate by considering all information available, for example, anatomical and external coordinates. According to this reasoning, crossing effects result from a usually adaptive strategy of multisensory integration rather than failure of spatial computations.

Other experimental results further substantiate the idea of weighted integration. In one experiment, uncrossed or crossed rubber hands were placed over participants’ hidden, real hands. The posture of the rubber hands was either congruent or incongruent with the real hands’ posture. Performance with crossed, real hands in a TOJ task improved when the rubber hands were uncrossed (Azañón and Soto-Faraco, 2007). Interestingly, this improvement was only observed when the movement of the real hand was coupled to the movement of the anatomically corresponding rubber hand, suggesting that visual influence on proprioceptive input depends on the degree to which the visual information about the body is coupled to the observer’s own actions. These results indicate that on-line visual information can be used adaptively, again suggesting that cross-modal integration is flexible on a short-term basis.

### SHORT TERM PLASTICITY: EFFECTS OF LEARNING

So far then, we have seen that the use of external spatial information for tactile localization seems mandatory, but that their weighting appears to be flexible. Yet, if weights are indeed adjustable, is it then really impossible to entirely ignore external spatial information, for example by giving it a zero weight? This question has been addressed by training sighted participants over ten sessions on different days in uncrossed and crossed hand TOJ (Craig and Belser, 2006). To allow learning, participants received feedback after every trial. TOJ performance with uncrossed hands remained unchanged throughout training. In contrast, performance with crossed hands improved over sessions, but asymptoted over the last 2–3 sessions, with a clear performance deficit still evident. These results imply that even extensive training does not enable us to entirely ignore external spatial coordinates. This controlled learning study has been complemented by studying musicians who frequently cross their hands when playing their instruments – professional drummers (Craig and Belser, 2006) and piano players (Kóbor et al., 2006). The study involving drummers did not find a difference between the crossing effect of musicians and non-musicians; the study involving pianists reported a reduction (but, importantly, not elimination) of the crossing effect. These findings are well in line with those of the controlled learning study, suggesting that, even if the weight of external coordinates can be reduced (as evident in the piano players), it does not seem to be possible to ignore them entirely.

### INEVITABILITY OF SPATIAL CODING

In attempting to answer the question whether remapping is mandatory, the TOJ paradigm suffers one drawback: the task is spatial in nature in that it requires participants to indicate which of two stimuli, defined by their location on the body, occurred first. To make a strong claim about the automaticity of tactile remapping, one should, however, show that the external location of a tactile stimulus affects performance even when all aspects of the experimental task are non-spatial. To address this concern, participants made color judgments about visual stimuli presented in the left and right space (Azañón et al., 2010a). Stimuli were preceded by a task-irrelevant tactile cue. In a first experiment, participants responded by pressing one of two buttons with a foot. In this situation, the color decision was improved when the tactile cue had been presented on the side of the visual stimulus. Importantly, this was the case even when the hands were crossed, suggesting that tactile attention was oriented to the external (already remapped) location of the hand. Because the foot response may have introduced a spatial aspect to the task, the experiment was repeated, but participants gave a verbal response. In this case, an effect of the tactile cue was still evident, though it was reduced. Finally, in a third experiment all visual stimuli occurred in one spatial location. They were either preceded by a tactile cue in some, but not in other trials. Blockwise, the cue was located near or far from the visual stimuli. Even in this situation, a spatial effect of the tactile cue was evident with crossed hands. Thus, this series of experiments suggests that touch is remapped even if spatial aspects are removed from the task. Nevertheless, spatial effects appear to be stronger if any aspect of the task bears spatial characteristics.

In sum, although the brain does seem to weigh spatial information for tactile localization in dependence of the current context, it appears to be reluctant to entirely discount any kind of information that is available. In the case of TOJ, the mandatory use of external coordinates leads to objective errors. This strategy may strike as counter-intuitive, if not maladaptive. However, similar reliance on different sources of information, and the attempt to integrate them into a common, sensible percept, has been evident in many other experimental situations as well. A very striking case is the Pinocchio illusion (Lackner, 1988). In this illusion, participants receive vibration to their biceps or triceps. This stimulation evokes the feeling that the arm is contracted or extended, respectively. If, at the same time, participants close their eyes and touch their nose, many experience their nose to be pushed into their head (for arm contraction), or to grow up to 30 cm long (for arm extension). Apparently, the brain tries to resolve the apparent conflict of the hand touching the nose while at the same time moving away from the head. Thus, rather than discounting incompatible information, the brain appears to prefer to integrate all available information in a seemingly meaningful manner. Other well-known examples of obligatory integration are the McGurk effect of visual and auditory speech perception (Mcgurk and MacDonald, 1976) and the ventriloquist illusion, in which we perceive the voice of an actor to originate from her doll (Bertelson, 1999).

## TOUCH AND THE REPRESENTATION OF THE BODY

**FIGURE 5:**
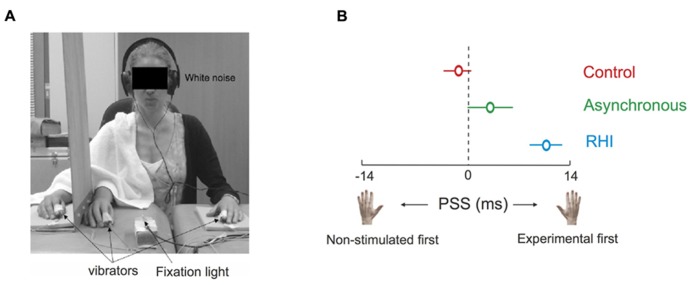
**Tactile processing during the rubber hand illusion (RHI).** When two tactile stimuli were applied in succession to each hand during the RHI on the right hand, TOJ responses were shifted toward the non-stimulated left hand (i.e., positive shifts of PSE). This suggests a change in the weight given to the processing of tactile information during the RHI, implying that ownership of an artificial body part is accompanied by consequences for the real body part. **(A)** a tactile TOJ task was presented to the participant during control trials (no stroking), asynchronous stroking of the rubber and the experimental hand, and during the RHI. **(B)** During the RHI, the tactile stimulus had to be presented to the experimental hand before the tactile stimulus was presented to the non-stimulated hand in order for them to be perceived as occurring at the same time, as compared to the asynchronous and control conditions. Figure modified from Moseley et al. (2008), Copyright (2008) National Academy of Sciences, USA.

The Pinocchio illusion illustrates how important touch is for the brain to create a representation of the body it commands. The importance of touch becomes evident in yet another intriguing illusion, namely the rubber hand illusion (RHI). Participants develop the feeling that an artificial hand belongs to themselves under the condition that their real (occluded) hand is touched in synchrony with touches they observe on the artificial hand (Botvinick and Cohen, 1998). Interestingly, the illusion additionally leads to a reduction of skin temperature in the limb involved in the illusion (Moseley et al., 2008). Moreover, when two tactile stimuli are applied in succession to each hand when the illusion is present in one hand, TOJ responses are shifted toward the non-stimulated hand, revealed by a change in PSS (Moseley et al., 2008). That is, in order for the two stimuli to be perceived as simultaneous, the tactile stimulus had to be applied to the experimental hand (the one that was previously stroked in synchrony with the rubber hand) before the tap is applied to the other hand. This change in PSS was interpreted as indicating a shift of processing priority toward the other, non-involved hand, in line with the idea that ownership of the rubber hand is accompanied by the disownership of the real hand (see **Figure 5**).

In recent years, it has been a prominent idea that the brain may represent tools in a manner similar to the body’s own limbs. In a famous study, macaque monkeys were trained to use a rake to retrieve raisins that were otherwise out of their reach (Iriki et al., 1996). Before training, neurons in the intraparietal sulcus responded to raisins when they were brought near the hand by an experimenter. After training, these neurons responded to raisins also when they were brought near the rake. These findings were interpreted to indicate that the rake was represented as belonging to the body (though see Holmes and Spence, 2004 for criticism of this study). If tools are assimilated to the body in some way after training, then the processing principles for the body should transfer to the processing of the tool. To test this idea, participants were asked to make TOJ of tactile stimuli presented to the end of sticks they were holding in their hands (Yamamoto and Kitazawa, 2001b; Yamamoto et al., 2005). In different conditions, participants held both their hands and the sticks uncrossed, or they crossed either their hands or the sticks. Note, that the hands remained uncrossed when the sticks were crossed; thus, although the tactile stimulus occurred at the crossed tool tip, the hand holding the stick and perceiving the stimulus remained uncrossed. Nevertheless, a comparable crossing effect was observed for both the hands-crossed (with tools uncrossed) and the tools-crossed (with hands uncrossed) conditions. Furthermore, in a fourth condition, participants crossed both hands and sticks, so that the sticks ended in the same hemifield as when hands and sticks were held uncrossed. In this situation, performance was almost as good as when hands and sticks were held uncrossed. The occurrence of a crossing effect thus seemed to depend on the position of the effector at which the stimulus was presented, that is, here, the sticks. In a follow-up study, the same authors used L-shaped rather than straight sticks (Yamamoto et al., 2005). Again, the crossing effect depended mainly on the tool tips being crossed in space, whereas the configuration of the hands and the tools were largely irrelevant. These results may indicate that, rather than the entire tool being incorporated to belong to the body, incorporation may be restricted to relevant parts of a tool (see also Holmes et al., 2004). For the sticks, this was their tip to which the touch was applied.

Interestingly, a similar effect of double crossing has been demonstrated without the use of tools, when stimuli were presented to the little fingers (Heed et al., 2012): In one condition, the hands were held uncrossed. In a second condition, the hands were crossed, and the little fingers changed side with the hands. In a third condition, the hands were again crossed, but the little fingers were crossed back into their normal hemifield. Performance with crossed-back fingers was improved compared to the normal crossed hands posture, but did not fully recover to uncrossed performance. Thus, like in the tool study by Yamamoto and Kitazawa, double crossing reduced the crossing effect. Once again, these results show that it is not crossing *per se* which causes the TOJ performance deficit, but rather the spatial configuration in which it results. The two studies may point to a general mechanism of touch localization: information about the posture of all body parts connected to the stimulated location may be integrated with their own weight. Integrating a tool would rely on the same mechanism, by simply adding some additional information about the spatial position of the tool to be integrated with the body’s postural information (cf. Heed et al., 2012). This proposal thus extends the idea of integration of anatomical and external-spatial information by adding information in both reference frames for several body parts.

## HANDS AND FINGERS

The fact that postural information about hands and fingers seems to be integrated in the crossed-back finger task suggests that touch on fingers and hands is remapped using a common external reference frame. Nevertheless, results of experiments concerning remapping for fingers and hands have not been unequivocal.

### SPATIAL REPRESENTATION OF THE FINGERS: EVIDENCE FOR SOMATOTOPIC CODING

Reports about patients with brain lesions have suggested that the brain entertains separate representations of the hands and the fingers. According to these reports, some patients can imitate finger movements, but not hand movements, whereas other patients present with the opposite deficiency pattern (Goldenberg and Karnath, 2006). With respect to touch localization, there are some tactile illusions which suggest that touch localization at the fingers does not take finger posture into account, at least when posture is atypical. For instance, when a participant crosses one finger over another and then touches an object with the two fingers, she will regularly perceive to be touching two rather than one object (termed Aristotle’s illusion, see Benedetti, 1985, 1988). This can be interpreted as a failure to integrate the atypical crossed posture of the fingers with tactile information, so that the location of the touch is processed as if the fingers were uncrossed. In the above example, the two skin sites touching the object when crossed (the outer sides of each finger) are usually non-adjacent when the hands are uncrossed. Thus, the brain tries to resolve the apparent conflict of two non-adjacent skin sites touching a single object by inducing the percept of two separate objects, rather than by integration of the fingers’ true posture. Intriguingly, these effects persist even after extensive training and only after a long period of adaptation with crossed fingers are participants able to remap the tactile stimuli to the correct finger (Benedetti, 1991). In a similar vein, a finger identification task (Haggard et al., 2006, though see Riemer et al., 2010) as well as tactile inhibition of return (Röder et al., 2002) were reported to be unaffected by finger posture. In yet a different approach, the direction of tactile motion presented to a single fingertip has been reported to be affected by a distracting stimulus with incongruent motion. However, this was only the case when the distractor was presented to a finger on the target hand, but not when target and distractor were delivered to different hands (Evans and Craig, 1991). This result supports the idea that orienting of tactile attention might be determined by somatotopic coordinates, before touch is remapped. Indeed, a subsequent study found similar interference effects when the fingers of the two hands were placed close together versus when they were placed farther apart, suggesting that external coordinates did not play a role for task performance (Evans et al., 1992, but see Zampini et al., 2005).

### SPATIAL REPRESENTATION OF THE FINGERS: EVIDENCE FOR EXTERNAL CODING

In sum, all these results suggest that touch to the hands and fingers may be represented differently in the brain. However, a different picture emerges from the TOJ task. In one study, a strong bias toward somatotopic coordinates was reported for a modified TOJ task in which participants judged the direction of movement of two tactile stimuli, one presented to the index and the other to the middle finger (de Haan et al., 2012). Note, that this instruction differs from the usual TOJ instruction to report the first stimulus, but that, nevertheless, temporal order is crucial to solve the task (as motion direction is defined by the order of stimuli). With crossed fingers, participants most of the time responded as if their fingers were uncrossed. However, a proportion of stimuli was reported in the correct direction, implying that remapping had probably taken place, but that remapped information was given little weight. In contrast, when participants were instructed to report the first stimulus (rather than a motion direction), a strong crossing effect was evident for crossed fingers (Heed et al., 2012). Thus, external coordinates appeared to play an important role in this version of the task.

Another study presented two tactile stimuli to two out of four possible fingers (the index and little fingers of the two hands; Badde et al., 2013). During half of the experiment, all fingers were uncrossed. In the other half, the index fingers were crossed, but the little fingers remained unchanged. When the two index fingers were crossed, and stimuli were presented to them, a large crossing effect was evident. However, when the first stimulus was applied to a crossed index finger, and the second stimulus was presented to an (uncrossed) little finger, the crossing effect was reduced. When the two little fingers were stimulated, crossing of the index fingers had no effect at all on performance. This finding suggests that the crossing effect depends on the specific posture of each involved body part (here, different uncrossed and crossed fingers).

Further evidence for the use of external coordinates in touch localization for the fingers has come from an experiment in which participants judged the direction of tactile motion over uncrossed fingers within a hand (Kuroki et al., 2011). Participants were first adapted to one direction for 10 s, with the fingers placed either crossed uncrossed, or vertically (that is, uncrossed but turned by 90°). Perceived motion direction was then assessed with horizontally aligned uncrossed fingers. Adaptation led to a motion direction after-effect opposite to the external direction of motion, independent of whether the fingers were crossed or uncrossed in the adaptation phase. Because the testing direction was orthogonal to motion adaptation in the vertical condition, no after effect was evident in that condition.

Summing up, findings concerning the spatial processing for touch on the fingers have been ambiguous. Neuropsychological findings suggest that differences between fingers and other body parts, especially the hands, do exist. How these differences pertain to tactile localization is not yet clear. Although some newer studies have suggested that touch is remapped for the fingers just like for other body parts, there is not yet an explanation for some phenomena, as for instance the Aristotle illusion, within such a framework.

## PAIN AND DISORDERS

The topics we have discussed potentially bear significance for clinical purposes. It is, therefore, exciting to see that the paradigms we have described in this paper have been used to investigate different types of disorders, as well as pain. The representation of the own body is a central aspect of pain processing. TOJ have proven useful to shed light on this relationship. Patients suffering from unilateral complex regional pain syndrome (CRPS), affecting one arm or hand, prioritized the processing of their unaffected hand. This was shown by a shift of the PSS of two tactile stimuli toward the unaffected hand, as compared to healthy controls (Moseley et al., 2009, 2012). Importantly, this effect reversed when the hands were crossed: patients now prioritized the hand that was located in the side of space affected by the pain syndrome, and not their affected hand (located in the “unaffected space”). Crossing did not affect the PSS in the healthy control group. This result suggests that the changes in tactile processing that accompany CRPS depend on the side of space in which the syndrome is located on the body, rather than on the affected limb *per se*. This finding implies that the perception of pain partly depends on its localization in external space. To investigate the neural underpinnings of this external spatial modulation, painful and non-painful stimuli were delivered to uncrossed and crossed hands of healthy participants (Gallace et al., 2011). For both types of stimuli, intensity ratings were lower in the crossed posture, and SEPs were reduced with crossed relative to uncrossed hands, starting at about 150 ms post-stimulus (referred to by the authors as N2, often referred to as N140 by others), whereas earlier processing was unaffected. Similarly, crossing effects in the standard TOJ task have been found to be comparable for painful and non-painful stimuli (Sambo et al., 2013). Thus, touch and pain processing show striking similarities. This, in turn, allows the formulation of hypotheses about which brain regions mediate the spatial processing common to both modalities. Given the crucial role of posterior parietal cortex in spatial transformations (Bolognini and Maravita, 2007; Azañón et al., 2010b; Takahashi et al., 2013), this region has been suggested as a prime candidate for the prevalence of external spatial reference frames also in pain processing (Sambo et al., 2013).

An involvement of parietal cortex has also been suggested for a disorder that has been described only recently, namely the body integrity identity disorder (BIID; Aoyama et al., 2012). Patients suffering from this disorder have a strong dislike for one part of their bodies, usually one limb, to the extent that they wish this limb were amputated. The etiology and mechanisms of this disorder have so far remained unclear, but the close relationship between body processing and touch localization have led to the hypothesis that basic processes in touch perception may be affected in BIID patients. To test this idea, BIID patients with dislike for the lower part of one of their legs made TOJ about two tactile stimuli, one on the unaffected, upper part, and one on the affected, lower part of the leg. Whereas the JND was comparable to healthy individuals (Schicke and Röder, 2006), the PSS was biased toward the affected limb. This was interpreted to reflect enhanced attention to the affected limb, consistent with the behaviorally evident over-concern that the patients display for this limb (Aoyama et al., 2012).

## SUMMARY, CONCLUSION, AND FUTURE DIRECTIONS

Our review has shown that TOJ have been an invaluable research tool for the investigation of tactile spatial processing and many topics beyond. In particular, we have highlighted the reliability and validity of the TOJ crossing effect as an indicator of the encoding of touch in external space. Nevertheless, several important questions remain.

The TOJ crossing effect is puzzlingly large. Yet, whereas remapping effects can be observed also in single stimulus paradigms (e.g., Overvliet et al., 2011; Badde et al., 2012), they are by an order of magnitude smaller than the two-stimulus TOJ effects. Elucidating the mechanisms behind the large TOJ effects will provide further knowledge of the current, and possibly new, models of tactile spatial processing and advance our understanding of somatosensory processing more generally. In this context, cognitive and computational modeling may be promising research tools by which the different theoretical accounts we have presented may be evaluated.

We have covered a number of broad topics related to touch and body processing. To begin with, several studies have attempted to characterize the timing of tactile remapping using crossed-hands paradigms (Yamamoto and Kitazawa, 2001a; Azañón and Soto-Faraco, 2008a; Heed and Röder, 2010; Overvliet et al., 2011; Soto-Faraco and Azañón, 2013). These studies suggest that tactile information is initially available in somatotopic space, but promptly transformed, within 100–200 ms, into external coordinates. However, although the results of these different studies appear largely consistent, the exact timeline of touch localization remains to be determined. Furthermore, recall that it has been suggested that the TOJ crossing effect arises from temporal imprecision (Kitazawa et al., 2008). Thus, drafting an adequate account of spatial touch processing may indeed require theoretical integration of spatial and time processing for this modality. It is, furthermore, of note that our understanding of the neural underpinnings of tactile spatial processing is limited, although first steps to map psychological function to brain regions and neural processes have been undertaken (e.g., Azañón et al., 2010b; Heed and Röder, 2010; Buchholz et al., 2011, 2013; Soto-Faraco and Azañón, 2013; Takahashi et al., 2013; Ruzzoli and Soto-Faraco, 2014).

Furthermore, a considerable number of TOJ studies have investigated the nature of the reference frames involved in touch localization. Whereas differences in tactile localization between blind and sighted individuals, as well as changes during ontogeny, suggest that visual coordinates are of particular importance in touch, the brain may consider additional reference frames in the definition of spatial information in touch. For example, whether spatial information related to movement planning and execution affect touch processing is currently unresolved. Similarly, most research has focused on a generalized visual coordinate system, leaving unresolved the specific representational code adopted by this reference frame. Even if it were strictly based on visual space, it could still be organized in many different forms, either in egocentric (e.g., retinotopically, trunk-, head or limb-centered) or allocentric (non-body related) space. How such representations are combined, and whether different contexts induce biases between them is still largely unknown.

A recent trend has been to conceptualize tactile remapping as an integrative process that weighs different pieces of spatial information from many sources, possibly according to current context like task requirements. This idea seamlessly connects to concepts of other domains of multisensory integration (Ernst and Banks, 2002). However, conclusive evidence that similar weighting processes are at work in different multisensory domains has not been made available. Clearly, such similarities bear the promise of discovering widely applicable, consistent processing principles across different sensory and cognitive domains. The concept of weighting is closely related to the intriguing topic of plasticity. If tactile remapping truly weighs reference frames depending on context, then the process of remapping must allow for rapid processing changes. The time and level of information required to allow these changes, their duration, and even the level of consciousness at which they might be processed are exciting topics for future research.

Finally, the relevance of touch to body processing has led to increasing interest in characterizing tactile behavior in different patient groups. For such investigations, two aspects are especially relevant for any experimental paradigm: on the one hand, it is desirable that results obtained from patients can be compared to a large body of knowledge obtained from a healthy population, so that conclusions about potential processing deficits can be drawn. On the other hand, any paradigm for use with patients should be easily applicable. TOJ fulfill both of these criteria, and they may thus be a good approach for further touch-related patient studies.

Taken together, we believe that the results obtained from the TOJ task highlight its utility in investigating tactile processing. This seemingly simple task has been used to build an extensive assembly of interconnected, widely relevant research findings, and the basis upon which new experiments can build is impressive. The TOJ task allows focusing on different aspects of behavior, including sensitivity, bias, and RT, allowing flexible use of the paradigm for many types of research questions. It will be delightful to see the paradigm used in future endeavors of psychological science.

## Conflict of Interest Statement

The authors declare that the research was conducted in the absence of any commercial or financial relationships that could be construed as a potential conflict of interest.

## Footnotes

1 To assess the 75% correct measure, one must measure the difference in SOA between chance responses (50%; that is, when left and right-hand first responses are perceived equally often) and 25% right-first responses for left-first trials (because 25% right-first responses are equivalent to 75% left-first responses). For right-first trials, one must, in analogy, measure the difference in SOA between 50 and 75% right-first responses. Thus, halving the distance between 25 and 75% gives the average of left-first and right-first SOA.
